# Development of Hepatocellular Carcinoma in a Patient with Chronic Hepatitis C 21 Years after Achieving a Sustained Virological Response to Interferon Therapy

**DOI:** 10.1155/2020/8824974

**Published:** 2020-10-14

**Authors:** Tasuku Hara, Tomoya Ohara, Masashi Taniguchi, Toshiaki Sakai, Kohei Oka, Naoto Iwai, Toshifumi Tsuji, Takashi Okuda, Toshiyuki Komaki, Junichi Sakagami, Keizo Kagawa

**Affiliations:** Department of Gastroenterology and Hepatology, Fukuchiyama City Hospital, Atsunaka-chou 231, Fukuchiyama, Kyoto 620-8505, Japan

## Abstract

A 77-year-old man with chronic hepatitis C (CH-C) infection, who achieved a sustained virological response (SVR) to interferon (IFN) therapy, was followed up regularly. Before IFN therapy, he did not have metabolic diseases, and the histological diagnosis of his chronic hepatitis was stage-3 fibrosis. After achieving SVR, the fibrosis-4 (FIB-4) index level dropped once but gradually increased. 21 years after SVR, hepatocellular carcinoma (HCC) was diagnosed by dynamic computed tomography. The HCC was 12 mm in diameter. The HCC was treated with radiofrequency ablation. CH-C patients with advanced fibrosis require long-term follow-up, even after achieving SVR.

## 1. Introduction

The hepatitis C virus (HCV) is a major cause of hepatocellular carcinoma (HCC) [1, 2]. The goal of therapy for chronic hepatitis C (CH-C) is the eradication of HCV. Until a few years prior, the only treatment strategy was based on interferon (IFN). In recent years, antiviral therapy against HCV has markedly improved, including the development of IFN-free direct-acting antivirals (DAA) [3]. Successful treatment with these drugs has resulted in a sustained virological response (SVR), defined as seronegative polymerase chain reaction for HCV-RNA six months after the cessation of therapy. An SVR has been reported to be associated not only with the improvement of hepatic inflammation and fibrosis but also with the inhibition of carcinogenesis [4, 5]. However, several studies have recently reported that HCC develops after an SVR to IFN or DAA in patients with CH-C [5–7]. Furthermore, there are reports of HCC developing over 20 years after SVR has been achieved [8–10]. We herein describe a male patient who developed HCC at the age of 77, 21 years after achieving an SVR to IFN. HCC was detected early as the cancer surveillance had been continued over the long term.

## 2. Case Report

**Figure 1:**
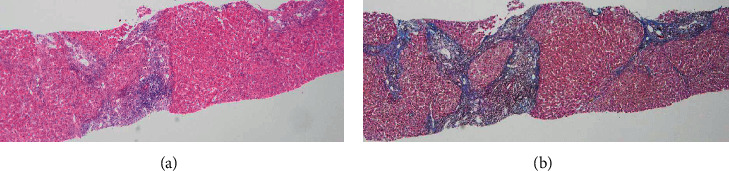
Histological findings of the liver biopsy specimen. (a) Moderate lymphocytic infiltrates in the portal areas of hepatic lobules with slightly piecemeal necrosis (hematoxylin and eosin staining; magnification, ×40). (b) Fibrous expansion of portal areas with occasional portal-to-portal bridging (= F3 fibrosis) (Masson's trichrome staining; magnification, ×40).

**Figure 2:**
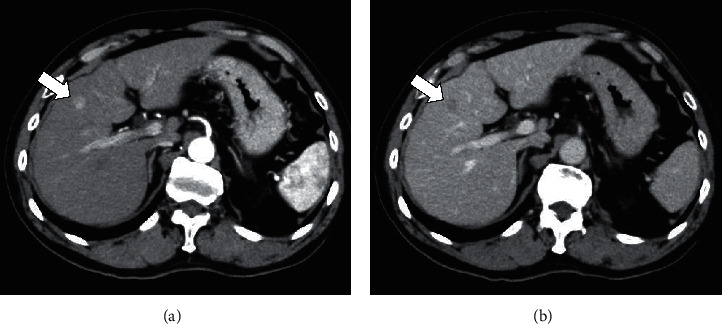
Contrast-enhanced CT showing the tumor (a) markedly enhanced in the arterial phase (b) followed by a relatively quick wash-out during the equilibrium phase (arrow).

**Figure 3:**
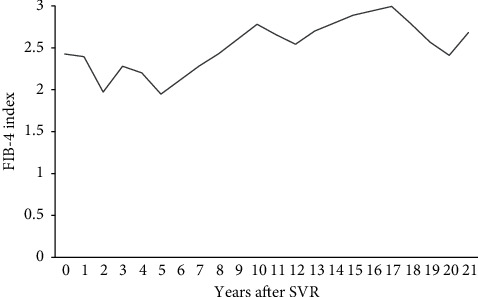
Time course of fibrosis-4 (FIB-4) index levels from SVR achievement to carcinogenesis.

The patient was a 77-year-old man with no chief complaints. He was a medical doctor specializing in interventional radiology and performed many angiograms of patients with viral hepatitis. He did not have metabolic diseases, including obesity and diabetes mellitus. He did not drink, but smoked 10 cigarettes a day. At the age of 41, he developed liver dysfunction and was diagnosed with CH-C several years later at a local hospital. HCV genotyping and viral load were unclear. At the age of 51, he was first referred to our hospital. He was treated with 6 million units (MU) of IFN-alpha monotherapy daily for 2 weeks, followed by 3 MU three times a week for 22 weeks, but showed no virological response. After that, he was treated with 10 MU of IFN-alpha 2b monotherapy daily for 2 weeks, followed by 6 MU three times a week for 22 weeks after histopathological confirmation of CH-C with stage-3 (F3) fibrosis (Figure 1). Completion of this therapy resulted in a negative test for HCV-RNA, and the achievement of SVR was at the age of 56 years. Thereafter, he underwent biannual blood tests and abdominal ultrasound (US) or computed tomography (CT). At the age of 77, 21 years after the SVR, CT identified a liver mass in segment 4 measuring 12 mm in diameter. In the arterial phase of enhanced CT, the tumor was markedly enhanced, followed by a relatively quick wash-out in the equilibrium phase (Figure 2). HCC was highly suspected based on the imaging features and medical history, including the response of HCV to therapy. Throughout the clinical course, the HCV-RNA remained negative; the hepatic steatosis was not observed by US and CT; the fibrosis-4 (FIB-4) index level dropped once, but gradually increased (Figure 3). He was admitted to our hospital for treatment. Laboratory data on admission are shown in Table 1. Protein induced by vitamin K absence or antagonist-II (PIVKA-II) level was elevated (60 mAU/mL). He tested negative for serum hepatitis B surface antigen, hepatitis B surface antibody, and hepatitis B core antibody. His body mass index was 23.6 kg/m^2^. Although surgery was recommended, the patient refused and underwent percutaneous radiofrequency ablation (RFA). At the last follow-up, 2 years after the treatment, the patient remained well and free of HCC recurrence.

## 3. Discussion

In this case, the patient developed HCC 21 years after achieving an SVR with IFN therapy. SVR has been reported to be associated not only with the improvement of hepatic inflammation and fibrosis but also with the inhibition of carcinogenesis [4, 5]. On the other hand, numerous patients have developed HCC after successful IFN or DAA therapy [5–7]. Sex (male), age (50 years or older), liver fibrosis (F3-4), and alpha-fetoprotein (10 ng/mL or more) are well-known risk factors for developing HCC after an SVR [6, 11]. Recently, it was reported that carcinogenesis is influenced by aging and lifestyle-related factors, such as alcohol consumption, metabolic disorders such as obesity and diabetes mellitus, and hepatic steatosis over 10 years after SVR [9, 12, 13]. Our patient had no risk factors for metabolic diseases for developing HCC after an SVR, but had a high risk due to his male sex, advanced age, and liver fibrosis.

Recently, several laboratory indices of liver fibrosis have been reported [14–16]. Vallet-Pichard et al. [16] reported that the FIB-4 index, calculated from laboratory values using AST (IU/L) × age (years)/platelet count (10^9^/L) × ALT (IU/L)^1/2^, is concordant with liver fibrosis assessed through pathological evaluation of liver biopsy specimens in patients with chronic HCV infection. Changes in the FIB-4 index have been shown to correlate with changes in the liver fibrosis stage over time in patients with HCV [17,18]. It is reported that the incidence of HCC development after an SVR is associated with the FIB-4 index level. Patients with FIB-4 index ≥3.25, indicating advanced liver fibrosis, after an SVR had a high risk for HCC incidence [19, 20]. A previous report showed marked improvement in liver fibrosis approximately 5 years after the SVR was achieved [21]. Regarding serum biomarkers, in patients achieving SVR, the FIB-4 index level decreased over a 10-year period, suggesting sustained regression of liver fibrosis [22]. In our case, before the start of IFN therapy, the patient was diagnosed with F3 fibrosis, and the FIB4-index level was 2.42 when the SVR was achieved. The FIB-4 index level dropped once, but gradually increased to 2.68, indicating progression of liver fibrosis at the time of carcinogenesis. Even if the FIB-4 index level is <3.25 after achieving an SVR, it may cause carcinogenesis if the FIB-4 index level increases again during follow-up. In terms of medical costs, it has been reported that HCC surveillance after an SVR is cost-effective for patients with cirrhosis, but not for patients with F3 fibrosis [23]. Previous reports span a maximum period of 10 years, so more long-term studies are needed.

Smoking has been suggested as a cofactor in the disease progression of various organs, including the liver, possibly through increased oxidative stress and inflammation. It has been reported that higher consumption of tobacco measured based on smoking pack-years was related to greater severity of fibrosis in patients with CH-C [24]. Furthermore, Balmaceda et al. [25] reported that smoking pack-years contribute to liver fibrosis after achieving an SVR. In our case, the patient had a habit of smoking 10 cigarettes a day for many years. It was possible that due to his smoking, liver fibrosis progressed again after achieving the SVR, and carcinogenesis occurred. It has been reported that smoking increases the risk of HCC incidence in patients with viral hepatitis [26]. However, it is not clear if smoking increases the risk of HCC independently in patients with HCV infection. Since the relationship between carcinogenesis and smoking after achieving an SVR is also unknown, further research is needed on the association between smoking and liver carcinogenesis due to HCV.

In recent years, antiviral therapy against HCV has markedly improved, especially treatment with DAA. The SVR achievement rate has increased to over 90% with DAA therapy [3]. However, it has also been reported that HCC develops after an SVR with DAA in patients with CH-C [7]. Early diagnosis of HCC at a curable clinical stage is necessary to improve the prognosis. Therefore, further studies are needed for appropriate method and interval of surveillance for HCC in patients who achieved SVR.

In conclusion, we report a case of HCC that developed 21 years after achieving an SVR. Since liver fibrosis plays an important role in the occurrence of HCC, monitoring of the FIB-4 index may be useful.

## Figures and Tables

**Table 1:** Laboratory findings on admission.

Variable	
White blood cells (/*μ*L)	4390
Red blood cells (10^4^/*μ*L)	396
Hematocrit (%)	39.1
Hemoglobin (g/dL)	13.0
Platelets (10^4^/*μ*L)	14.1
PT-INR	0.96
PT (%)	107.5
Total protein (g/dL)	6.3
Albumin (g/dL)	4.1
C-reactive protein (mg/dL)	0.05
BUN (mg/dL)	16
Creatinine (mg/dL)	0.88
T-chol (mg/dL)	173
Glucose (mg/dL)	92
HbA1c (%)	5.8
Total bilirubin (mg/dL)	0.7
AST (IU/L)	17
ALT (IU/L)	12
ALP (IU/L)	207
GGT (IU/L)	17
AFP (ng/mL)	19
PIVKA-II (mAU/mL)	60
CEA (ng/mL)	1.3
CA19-9 (U/mL)	3
HCV Ab	Positive
HCV-RNA	Negative
HBsAg	Negative
HBsAb	Negative
HBcAb	Negative
FIB-4 index	2.68

PT, prothrombin time; PT-INR, prothrombin time-international normalized ratio; BUN, blood urea nitrogen; T-chol, total cholesterol; AST, aspartate aminotransferase; ALT, alanine aminotransferase; ALP, alkaline phosphatase; GGT, gamma-glutamyl transpeptidase; AFP, alpha-fetoprotein; PIVKA-II, protein induced by vitamin K absence or antagonist-II; CEA, carcinoembryonic antigen; CA19-9, carbohydrate antigen 19-9; HCV Ab, hepatitis C virus antibody; HBsAg, hepatitis B surface antigen; HBsAb, hepatitis B surface antibody; HBcAb, anti-hepatitis B core antibody; FIB-4 index, fibrosis-4 index.

## Data Availability

No data were used to support this study.
